# The bite force and craniofacial morphology in patients with acromegaly: A pilot study

**DOI:** 10.4317/medoral.18984

**Published:** 2013-08-29

**Authors:** Duygu Karakıs, Banu Aktas-Yılmaz, Arife Dogan, Ilhan Yetkin, Bulent Bek

**Affiliations:** 1 Research Asistant, Department of Prosthodontics, Faculty of Dentistry, Gazi University, Ankara, Turkey; 2 Research Asistant, Department of Endocrinology and Metabolism, Faculty of Medicine, Gazi University, Ankara, Turkey; 3 Professor, Department of Prosthodontics, Faculty of Dentistry, Gazi University, Ankara, Turkey; 4 Professor, Department of Endocrinology and Metabolism, Faculty of Medicine, Gazi University, Ankara, Turkey

## Abstract

Objectives: Acromegaly is a metabolic disorder caused by increased growth hormone secretion. As a consequence of acromegaly some typical craniofacial morphology changes appear. This pilot study was conducted to compare the bite force and the characteristic size and shape of the craniofacial components of acromegalic patients with the healthy Turkish individuals. In additon, the correlations between bite force and craniofacial morphology of patients with acromegaly and control individuals were evaluated. Study Design: The maximum bite force of the participants was recorded with strain-gage transducer. Lateral x-ray scans were made under standard conditions, in centric occlusion. On cephalograms, the linear and angular measurements was performed. Results: Patients with acromegaly showed increased anterior and posterior total face height, ramus length, width of frontal sinuse, gonial angle and a negative difference between maxillary and mandibular protrusions. In addition, females with acromegaly showed larger lower anterior face height and sella turcica, decreased facial angle, increased mandibular plane angle. The cephalometric measurements, except one did not showed correlation with the bite force in acromegalic patients. In control group, significant correlations were observed between anterior total face height and anterior lower face height, mandibular plane angle and gonial angle. Conclusions: The greater changes were observed in the mandible. The maximum bite force of patients with acromegaly showed no difference from healthy individuals. The non-significant difference of bite force between healthy participants and acromegalic patients provide important information for dental treatment and prosthetic rehabilitation of acromegalic patients.

** Key words:**Acromegaly, bite force, cephalometric analysis, mandibular prognathism.

## Introduction

Acromegaly results from the chronic hypersecretion of growth hormone (GH), usually caused by an adenoma of the pituitary gland (1,2). It is a rare condition with an estimated prevalence of around 60 per million and an annual incidence of 3-4 patients per million (3). It is characterized by the somatic disfigurement, mainly involving face and extremities, and metabolic manifestations that are attributable to high serum concentrations of both GH and insulin like growth factor-1 (IGF-1) (2). The somatic effects include stimulation of growth of many tissues, such as skin, epithelial tissues, connective tissues, cartilage, bone, viscera, cardiovascular and pulmonary system (4). Under the influence of the GH and IGF-I, new bone formation leads to characteristic alterations in the craniofacial morphology, such as the mandibular prognathism, diastemas, malocclusion, frontal bossing and hypertrophy of sinuses, especially the frontal sinuses (2,5-8). Acromegaly is a slowly progressive disease and these craniofacial morphologic changes occur gradually, for this reason the diagnosis may be difficult or delayed (9). Therefore, the accurate identification of the skeletal, dental and structural abnormalities is important for early diagnosis of the disease. Besides, the determination of the size and direction of enlargement of the mandible and maxilla is useful to set an appropriate dental treatment plan for the patients with acromegaly.

The influence of craniofacial morphology on the masticatory system have previously been reported (10,11). The bite force is also used as an indicator of the masticatory system (12). It has been shown that the craniofacial morphology had a strong relation with the bite force (12-15). Furthermore, it has been stated that the patients with long face had lower bite force than those with short or normal face (13). In other words, strong bite force is seen in individuals with more anteriorly inclined mandible, a smaller anterior and greater posterior facial height and smaller gonial angle (13,16,17). Based on these observations, similar pattern between craniofacial morphology and bite force may be seen in patients with acromegaly who have prominent craniofacial characteristics. Besides, the reveal of the effect of enlarged mandibula as seen the most remarkable sign of acromegalic patients on bite force may be useful for better understanding of the relation of bite force and size of mandible. The correlation between facial morphology and bite force is also considered as an important factor in individuals with orthodontic treatment and orthognathic surgery (18-20). The purpose of this pilot study was to compare the bite force and craniofacial components of acromegalic patients with the healthy Turkish subjects. Furthermore, it was evaluated whether there was a correlation between the bite force and craniofacial morphology of the patients with acromegaly and healthy individuals.

## Material and Methods

-Participants

This pilot study was conducted on 11 patients with acromegaly, including 5 male and 6 female. The experimental protocol was approved by the Ethics Committee of Gazi University (Process# 092). All paticipants received a written explanation of the study, and informed consent was obtained from each person in the beginning of the study. The diagnosis of acromegaly was made using clinical examination and biochemical assessment that included high serum concentrations of GH and IGF-1; and failure of normal supression of serum GH levels following the administration of 75 gram oral glucose. Radiological assesment was made with magnetic resonation imaging (MRI) of the pituitary gland.

The participants in control group were not exposed to the x-rays due to the ethical reasons. The results of the control group obtained from a thesis (written by the researcher; DK) that evaluated the corelation between the bite force and the cephalometric measurement of Turkish healthy individuals in the normal standards for the skeleton. The control group comprised of 11 partici-pants including 5 male and 6 female.

-Craniofacial analysis

Lateral x-ray scans were made under standard conditions, in centric occlusion with the control of head position. On cephalograms, the linear and angular measurements were performed. Linear measurements included anterior and posterior cranial base length (S-N, S-Ba); ramus length (Ar-Go); mandibular corpus length (Go-Me); length of the maxilla (Ans-Pns); anterior and posterior face height (N-Me,S-Go); lower face height (Ans- Me); width of frontal sinus (F1-F2); anterioposterior dimension of sella turcica (S1-S2). Angular measurements are the antero-posterior position of the maxilla relative to the anterior cranial base (SNA); the antero-posterior position of the mandible relative to the anterior cranial base (SNB); the magnitude of the skeletal jaw discrepancy (ANB); cranial base angle (N-S-Ba); saddle angle (N-S-Ar); articular angle (S-Ar-Go); gonial angle (Ar-Go-Me), vertical jaw relationship (Ans-Pns/Go-Me); mandibular plane angle (FH/Go-Me); angle of convexity (N-A-Pg); slope of the palatal plane (FH/Ans-Pns).

-Recording of bite force 

Maximum bite forces were measured from each side of the dental arch using two miniature strain-gauge transducers with stain-less-steel cases (Model VLPB, Load CellCentral, Monroeton, PA, USA). Two transducers were placed bilaterally on a flat metal arch, and the button of the strain-gauge transducer was in contact with the flat metal arch. The transducers were fixed with plaster (Betasan, Kocaeli, Turkey) to the metal arch. The metal arch and transducers were further covered with a disposable latex finger coating to avoid contamination during measurements. Each transducer had a height of 4 mm and a diameter of 12 mm; in these applications, transducers reached a height of 6 mm. The bite force was detected as a two-channel signal from each side with a biosignal acquisition device designed by Kardiosis (Tepa, Kardiosis, Ankara, Turkey). The force signals were monitored online and then measured on a PC screen, using a specific software program developed by the same company. The transducers were calibrated by loading them with known force values.

During the test, participants were seated in an upright position with the head in a natural posture to keep the Frankfort plane approximately parallel to the floor. The transducers were also maintained parallel to the Frankfort plane. Initially, the bilateral transducers were positioned on the metal plate and were placed between the first molar teeth on both sides. The participants were asked to clench their teeth as forcefully as possible three times. The highest value of each clenching was recorded as kilogram-Watt (kgW), and the mean value of the three highest clenching was considered the participant’s maximum bite force. The sum of the right and left bite force values was considered to be the maximum bite force.

The measurements were carried out twice with 2 weeks intervals and they revealed no differences. Both of the cephalometric and bite force measurements was evaluated by the same examiner.

-Statistical analysis

All data were analyzed using the Statistical Package for the Social Sciences version 16.0 (SPSS Inc, Chicago, IL, USA). Mann-Whithney U test was used to compare differences in the bite force and cranifacial dimensions between the patients with acromegaly and control group. The Pearson correlation coefficient was performed to assess correlations between bite force and cranifacial measurements in patients with acromegaly and control group. Differences at the 5% level of probability were considered statistically significant.

## Results

The linear and angular cephalometric measurements and the bite force of female and male volunteers are shown in  Table 1, Table 2, respectively. In comparison with the controls, the patients of both gender revealed enlargement in anterior and posterior total face height (N- Me, S-Go), ramus length (Ar-Go), width of frontal sinuses (F1-F2). The angular measurements of patients exhibited an enlarged gonial angle (Ar-Go, Go-Me), a negative difference between maxillary and mandibular protrusions (ANB). When compared with the controls, females with acromegaly showed larger lower anterior face height (Ans-Me) and Sella Turcica (S1-S2), decreased facial angle (N-A/A-Pg), increased mandibular plane angle (Go-Me/FH). The other cephalometric measurements showed no statistical significant differences between male individuals (P<0.05).

**Table 1:**
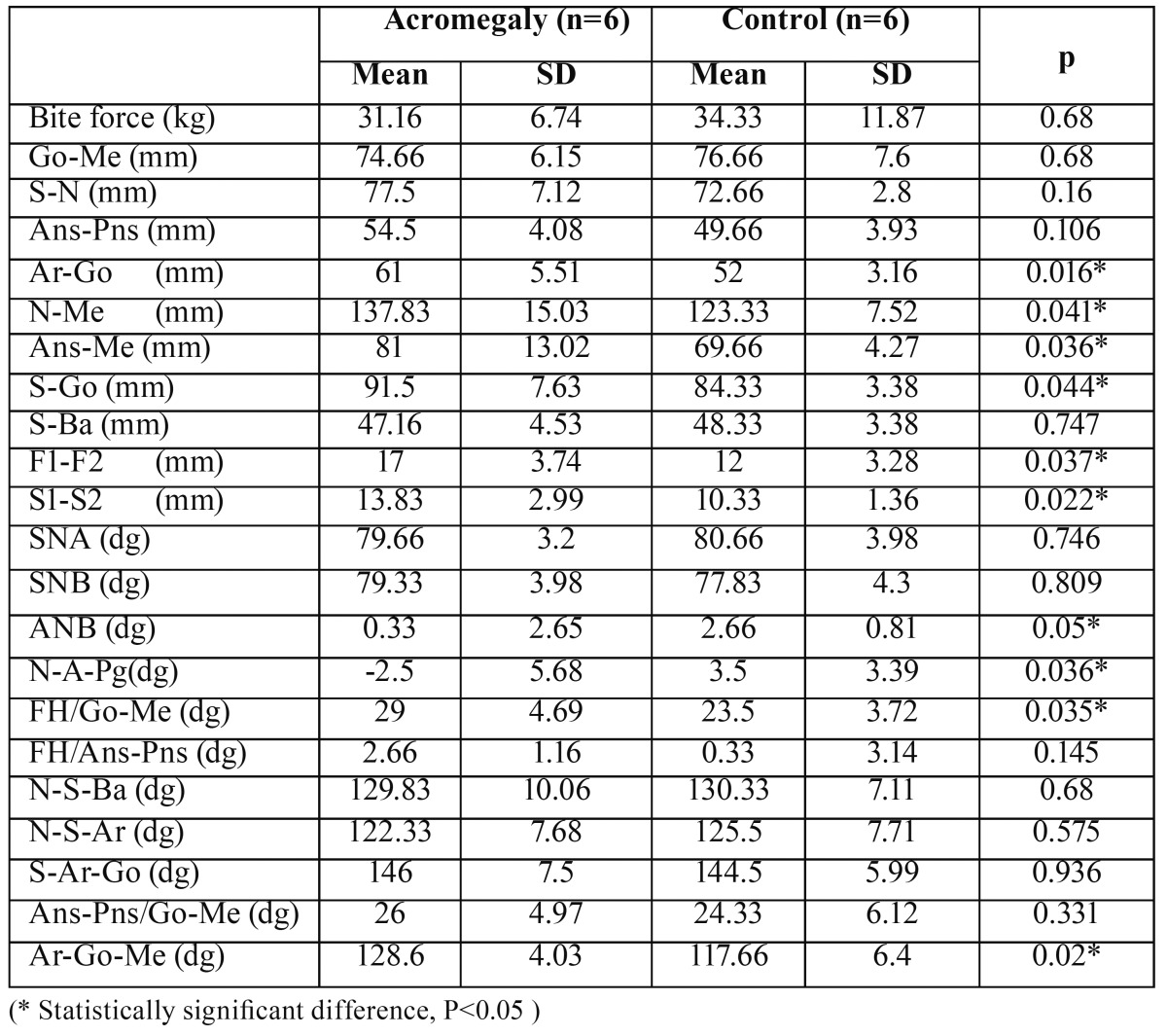
Bite force and cephalometric measurements of female acromegalic patients and controls.

**Table 2:**
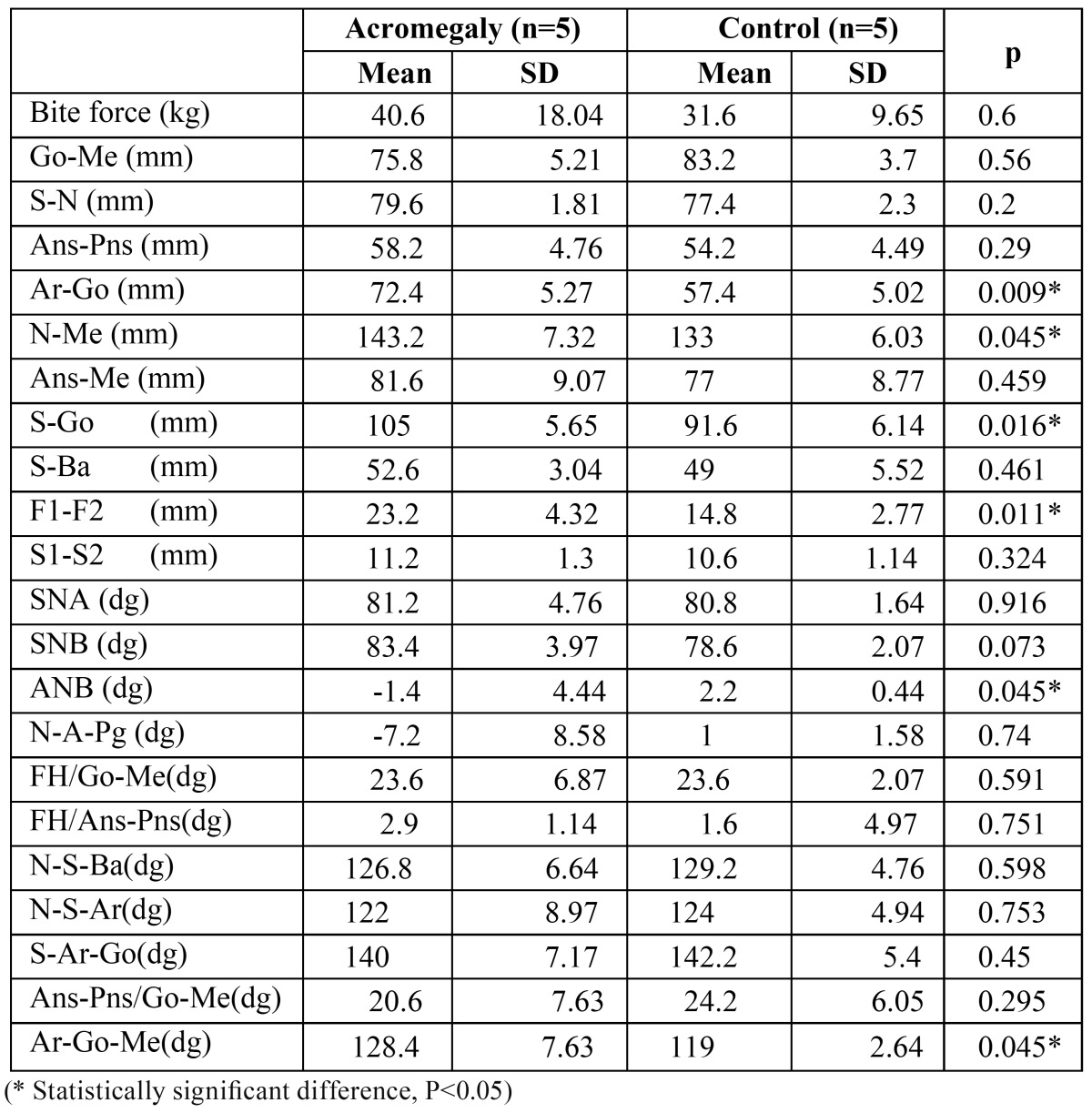
Bite force and cephalometric measurements of male acromegalic patients and controls.

Maximum bite force had no significant difference between healthy individuals and patients with acromegaly in both of female and male (P>0.05; p=0.68, p=0.6, respectively).

Correlation among the factors are shown in  Table 3. Only one of the linear cephalometric measurements (posterior face height) was significantly related to bite force in patients with acromegaly (r = - 0.658, p= 0.028). No correlation was observed between the bite force and other cephalometric measurements in acromegalic patients (P>0.05). In control group, significant correlations were found between the bite force and two linear measurements and two angular measurements, which are anterior total face height and anterior lower face height; mandibular plane angle and gonial angle (P <0.05).

**Table 3:**
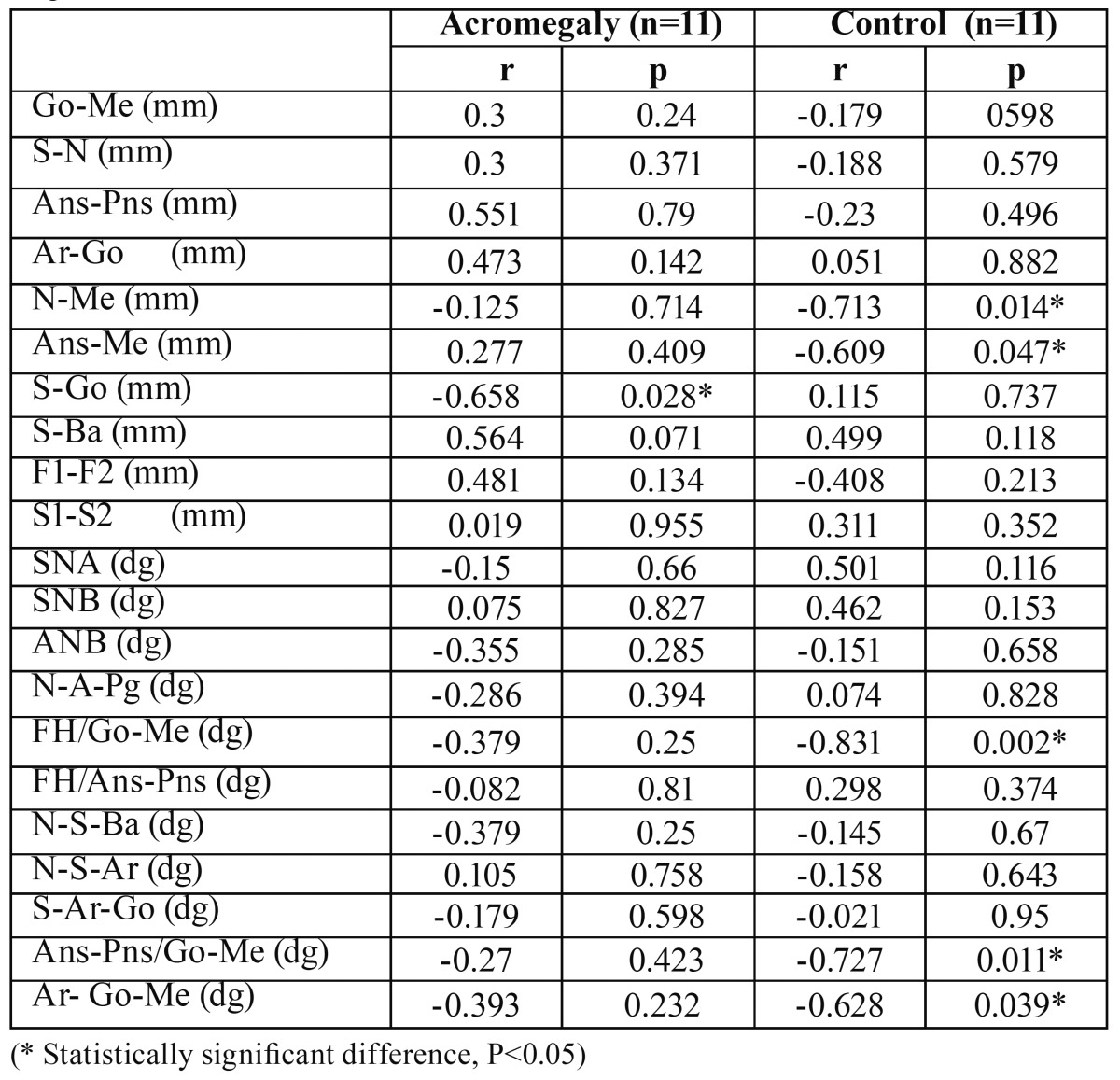
Correlation between bite force and cephalometric measurements in acromegalic patients and controls.

## Discussion

Typical craniofacial changes of patients with acromegaly have been described by many authors. It is generally believed that acromegalic patients are characterized by a Class 3 malocclusion associated with a severe mandibular prognathism, increased gonial angle and typical alteration of the facial features (5-8). In the present study, the ANB angle of acromegalic patients showed statisticaly significant difference from healthy individuals for both of the gender. However, the mean ANB angle of females was not negative and did not indicate Class 3 malocclusion, in addition one of the female patient had skeletal Class 2 malocclusion. This result is in good agreement with the findings of the study by Dostolava et al. (6) in which acromegalic patients with Class 2 malocclusion were reported. In the current study, there was increased gonial angle in both of gender that indicates tendency to posterior movement of the mandible. In addition, females had decreased facial angle (N-A/A-Pg) and increased mandibular plane angle (Go-Me/FH) which shows that females have more tendency for severe mandibular protrusion and posterior rotation of mandible.

The linear measurements revealed increased anterior and posterior total face height, enlargement of the ramus length and width of the frontal sinuses for both of the gender. Dostalova et al. (6) have evaluated cranial abnormalities in patients with acromegaly and they have exhibited increased facial height; a negative difference between maxillary and mandibular protrusions; and enlarged lower part of the gonial angle. The patients have also exhibited enlargement of all parts of the neurocranium and orofacial bones except maxilla (6). These findings are in agreement with that of our study in which non-significant changes in the position of the maxilla were observed. Kunzler and Farmand (21) have examined cephalometric parameters in 31 acromegalic patients of both gender and in 21 healthy individuals. In accordance with the results of the present pilot study, they have reported that the individuals had increased mandibular protrusion, increased length of the mandible, altered sagittal jaw relations and so no differences in the position of maxilla (21). Takakura et al. (5) have evaluated the most characteristic craniofacial skeletal differences in patients with acromegaly. In contrast to our results, they have stated that females showed bimaxillary alveolar protrusion. On the other hand, they have found increased anterior face height, enlarged ramus and downward mandibular advancement in the acromegalic patients of both gender, which was in accordance with our study. They have found enlargement of sella turcica and sinus frontalis in both gender however in the present study only females showed enlargement in that measurements (5).

While some previous studies reported that pressure of a large tongue or trusting forward affects of tongue led to enlargement of the maxillary alveoar bone and mandibular bone in patients with acromegaly (1,2), most of the studies reported no enlargement of maxilla as a facial characteristic feature of acromegalic patients (6,7,21-23). Although it is unclear whether the protrusion of maxilla is a characteristic sign of the acromegalic patients, it is obvious that the enlargement of mandible is more prominent than maxilla. In the current study, the mandible was mostly affected, showing greater enlargement in the ramus than body of the mandible. This result is in agreement with the study performed by Dostalava et al. (6).

Clinical studies have indicated a correlation between craniofacial morphology and the bite force (16,24-26). It has been suggested that bite force reflected the geometry of the lever system of the mandible (27). Greater mechanical advantage and relatively stronger bite forces are associated with more anteriorly inclined mandible, smaller anterior facial heights, greater posterior face height as well as a smaller gonial angle (16,24,26,28). In accordance with these results, in the present study, the healthy individuals showed negative correlation between bite force and anterior total face height, lower anterior face height, gonial angle and mandibular plane angle. However, patients with acromegaly exhibited correlation between only one of the cephalometric measurement (posterior face height) and bite force. On the other hand, there was no significant difference in the bite force between patients with acromegaly and control group in both of gender. When compared with healthy individuals, the patients with acromegaly had increased anterior total face height and gonial angle that is associated with decreased bite force. Besides, they had malocclusion and greater posterior facial height that is related to increased bite force. The non-significant difference of bite force between control group and acromegalic patients or absence of correlation between the bite force and facial morphology in acromegalic patients may be explained by different factors. Primarily, bite force magnitude depends on the size and the length of the moment arm of the jaw muscles which is modified by craniofacial morphology (11,29). It has been stated that bite force is a result of the geometric arrangement of the lever system of the jaw (30). In acromegalic patients typical skeletal enlargement is apparent however the geometric arrangement of these skeletal enlargement of craniofacial morphology might negatively affects the mechanical advantage of mandible and thereby bite force. On the other hand, although Freda et al. (31) stated that muscle mass in acromegaly patients did not differ from control values, the effect of this disease on muscle size and strength is not clear. Therefore, further studies are required to evaluate the effect of the acromegaly on the strength and size of the masticatory muscles and reveal the biomechanical aspects of the craniofacial enlargement in patients with acromegaly.

The correlation between the bite force and facial morphology in healty individuals also should be considered in orthognathic patients. Bite force has been used to evaluate masticatory function in patients before and after orthognathic surgery. Throckmorton et al. (20) stated that anterior and posterior facial height were both strongly correlated with maximum bite force and reflected the assignment of surgical procedures. Therefore, the determination of effective craniofacial measurements that best predict maximum bite forces is important in orthognathic patients.

## Conclusion

The main limitation of this pilot study was the small number of the participants. The patients with acromegaly showed increased anterior and posterior total face height, enlargement of the ramus length and width of frontal sinus, enlarged gonial angle, and a negative difference between maxillary and mandibular protrusions. The greater changes were observed in the mandible. The maximum bite force of patients with acromegaly showed no difference from healthy subjects. The craniofacial changes in patients with acromegaly are very important for early detection of disease, therefore dentist, orthodontists and maxillofacial surgeons should be well aware of disease. Besides, the analysis of mentioned characteristic craniofacial changes and non-significant difference of bite force between healthy individuals and acromegalic patients may contribute to better orthodontic, surgical and dental management of acromegalic patients.
